# Global Reprogramming of Apoptosis-Related Genes during Brain Development

**DOI:** 10.3390/cells10112901

**Published:** 2021-10-27

**Authors:** Wei Jiang, Liang Chen, Sika Zheng

**Affiliations:** 1 Department of Quantitative and Computational Biology, University of Southern California, Los Angeles, CA 90089, USA; jian227@usc.edu; 2 Division of Biomedical Sciences, University of California, Riverside, CA 92521, USA

**Keywords:** epigenome, *Bax*, *Bak1*, *Casp3*, *Casp7*, *Casp6*, *Casp8*, *Casp9*, *Apaf-1*, cytochrome c

## Abstract

To enable long-term survival, mammalian adult neurons exhibit unique apoptosis competence. Questions remain as to whether and how neurons globally reprogram the expression of apoptotic genes during development. We systematically examined the in vivo expression of 1923 apoptosis-related genes and associated histone modifications at eight developmental ages of mouse brains. Most apoptotic genes displayed consistent temporal patterns across the forebrain, midbrain, and hindbrain, suggesting ubiquitous robust developmental reprogramming. Although both anti- and pro-apoptotic genes can be up- or downregulated, half the regulatory events in the classical apoptosis pathway are downregulation of pro-apoptotic genes. Reduced expression in initiator caspases, apoptosome, and pro-apoptotic Bcl-2 family members restrains effector caspase activation and attenuates neuronal apoptosis. The developmental downregulation of apoptotic genes is attributed to decreasing histone-3-lysine-4-trimethylation (H3K4me3) signals at promoters, where histone-3-lysine-27-trimethylation (H3K27me3) rarely changes. By contrast, repressive H3K27me3 marks are lost in the upregulated gene groups, for which developmental H3K4me3 changes are not predictive. Hence, developing brains remove epigenetic H3K4me3 and H3K27me3 marks on different apoptotic gene groups, contributing to their downregulation and upregulation, respectively. As such, neurons drastically alter global apoptotic gene expression during development to transform apoptosis controls. Research into neuronal cell death should consider maturation stages as a biological variable.

## 1. Introduction

Apoptosis is a ubiquitous regulated cell death pathway in multi-cellular organisms, essential for development, tissue homeostasis, and immune system functions [1]. Various physiological or pathological stimuli can induce apoptosis. Depending on the origin of a triggering stimulus, the signaling cascades leading to apoptosis are commonly attributed to the intrinsic (mitochondrial) pathway and the extrinsic (death receptor) pathway, though the two pathways can influence one another [2,3,4,5]. Both pathways converge on activation of executioner caspases that cause cellular structure breakdowns and DNA fragmentation [6,7,8].

The significance of apoptosis in the mammalian nervous system is well documented [9,10]. Dysregulated apoptosis is implicated in a wide spectrum of neurodevelopmental disorders and neurodegenerative diseases. During development, apoptosis plays an essential role in establishing appropriate neuronal cell numbers and forming robust neural circuits [11,12]. For example, peripheral neurons depend on target-derived trophic factors for survival and without them undergo apoptosis [13,14]. As such, the formation of neural circuits is a selection process through apoptotic control.

Despite the importance of apoptosis for neuronal development, neurons reduce in apoptotic competence as they mature [15,16]. In the peripheral nervous system, withdrawal of neurotrophins drastically induces apoptosis in developing neurons but barely affects mature neurons [14,17,18]. In the central nervous system, the vulnerability of post-mitotic cortical neurons to apoptosis is much lower than cortical progenitors. Apoptosis occurs more frequently in neural stem and progenitor cell-enriched ventricular and subventricular zones than in cortical plates consisting of post-mitotic neurons [19,20,21,22,23,24]. In adult brains, mature neurons become less susceptible to various apoptotic stimuli, including ionizing radiation and hypoxic stress [25,26,27,28]. The attenuation of neuronal apoptosis during development prevents mature neurons from prematurely committing apoptotic cell death, allowing uninterrupted neuronal survival throughout an organism’s life, which is essential for fitness and survival.

Accordingly, neurons must transform how they regulate apoptosis during development. However, the molecular mechanisms transforming neuronal apoptosis regulation during development are less clear. The expression control of apoptotic genes is presumably at the center of this regulatory program. To date, 1923 genes have been annotated by the Gene Ontology Consortium as involved in or regulators of apoptosis. This regulatory nexus formulates the check and balance before a cell undergoes apoptosis. Altered expression of apoptotic genes is widely implicated in cancer development and other disease progression [29,30]. With varied expression of apoptosis-related genes (herein also termed apoptotic genes), neural progenitors and immature and mature post-mitotic neurons can respond differently to apoptotic stimuli.

While the role of apoptosis in neuronal development has been extensively studied and the contributions of individual apoptotic genes have been demonstrated in piecemeal with genetically modified mice, the question remains as to how the neuronal regulatory program of apoptotic genes as a whole is shaped during neuronal development. In this study, we comprehensively examined the in vivo temporal regulation of all known apoptosis-related genes in multiple brain regions during mouse development from embryonic day 10.5 (E10.5) to postnatal day 0 (P0). This period coincides neurogenesis and neuronal differentiation, and largely excludes gliogenesis. We determined to what extent and with what patterns apoptotic genes are regulated during neuronal development.

We further investigated whether chromatin-based gene regulation could be the underlying regulatory mechanism. Epigenetic regulation, in cooperation with transcription factors, determines the transcriptional output of gene expression [31,32]. A major epigenetic regulatory mechanism is histone modification. Histone H3 tri-methylation on lysine 4 (H3K4me3) and lysine 27 (H3K27me3) are a pair of important modifications at and around gene promoters that activate and repress gene expression, respectively [33,34,35,36]. H3K4me3 modification is generated by specific histone methyltransferases (e.g., UTX, SETD1A) and is highly enriched near transcription start sites of active promoters [37,38]. H3K27me3 deposited by histone methyltransferases (e.g., EZH2) of PRC complexes is a hallmark of polycomb group-mediated transcriptional silencing [39]. These histone modifications may serve as an upstream regulation layer to orchestrate the global chronological expression changes of apoptotic genes, but their contributions have not been studied. Knowledge about the developmental reprogramming of the apoptotic gene expression and the underlying regulatory mechanisms is essential to understanding the activity and regulation of the apoptosis pathway in neural development and diseases.

## 2. Materials and Methods

### 2.1. Definition of Apoptosis-Related Genes

We downloaded GO terms of mouse genes from the Ensembl BioMart https://uswest.ensembl.org/index.html (accessed on 1 October 2021). Genes with GO terms including the keyword “apopt” (e.g., apoptosis, apoptotic) were considered, resulting in a total of 1923 apoptosis-related genes. These GO terms are listed in Table S1. Based on the assigned GO terms, 1282 of 1923 apoptotic genes are involved in either positive or negative regulation of cellular apoptosis. To analyze regulation direction, genes solely involved in positive regulation of apoptosis were defined as pro-apoptotic genes, and those in the negative direction were defined as anti-apoptotic genes. The 153 genes involved in both directions were excluded for analyses involving regulation direction. The remaining genes have no information available regarding regulation direction in apoptosis.

### 2.2. Experimental Data Acquisition

Datasets of mRNA expression (mRNA-seq) and histone ChIP-seq were downloaded from the mouse Encyclopedia of DNA Elements (ENCODE) project (http://encodeproject.org) (accessed on 1 October 2021). These corresponded to three brain tissues (forebrain, midbrain, and hindbrain) of the Mus musculus C57BL/6 strain, and eight developing ages (embryonic days 10.5, 11.5, 12.5, 13.5, 14.5, 15.5, and 16.5, as well as to postnatal day 0). The histone modification marks included H3K4me3 and H3K27me3 for the same brain regions and developing ages. Data from the two biological replicates of each experimental configuration were averaged.

### 2.3. RNA-Seq Data Analysis

Processed gene quantification data of two biological replicates from the forebrain, midbrain, and hindbrain tissues were downloaded from ENCODE. Expressed apoptosis-related genes were defined using the following cutoffs: transcripts per million (TPM) ≥ 1 in at least one-third of data points and maximum TPM ≥ 5 among all data points. The Z-score normalization on log_2_(TPM + 1) was performed before the hierarchical clustering analysis using the UPGMA agglomeration method. Two major clusters were labeled as the upregulation cluster and the downregulation cluster based on their overall chronological trends. The same analyses were performed for data from the three regions separately.

### 2.4. ChIP-Seq Data Analysis

For the ChIP-seq data, “bed narrowPeak” files containing the peaks of signal enrichment and “bigwig” files containing signal p-values were obtained from ENCODE. For each sample, the file containing either replicated peaks or pseudoreplicated peaks was downloaded based on the default recommendation from ENCODE. Defined by ENCODE, replicated peaks are peak calls from pooled replicates and pseudoreplicated peaks are calls from two analysis partitions. Significant peaks were annotated with the ChIPpeakAnno package [40] using mm10 Ensembl genes as references. Peaks residing from −2 kb to 2 kb around a transcription start site (TSS) were defined as histone modification signals over the promoter region. Peak intensity data were obtained from the bed narrowPeak files. For a promoter overlapping with multiple peaks, the peak with maximum intensity was taken for further analysis. The ChIP-seq tracks were visualized using the UCSC genome browser.

### 2.5. Regression Fit between RNA-Seq (or ChIP-Seq) Data and Ages

Linear regression models were fit between expression levels (log_2_(TPM + 1)) of each gene in the upregulation or downregulation clusters and the considered age time points (P0 was coded as day 22). For the H3K4me3 or H3K27me3 ChIP-seq data, linear regression models were fit between the promoter peak intensity and ages. The age coefficient for each gene was denoted as β_Ex_, β_K4_, and β_K27_ for expression, H3K4me3, and H3K27me3 signals respectively. The coefficient represents the change of the expression or the histone modification when the age advances one day.

### 2.6. KEGG Apoptosis Pathway

The classical apoptosis pathway (entry: hsa04210) was acquired from KEGG (Kyoto Encyclopedia of Genes and Genomes, https://www.genome.jp/kegg/, accessed on 1 October 2021). and then replotted for visualization purposes. Genes from the upregulation cluster in forebrain, midbrain, or hindbrain regions were labeled as “upregulation cluster” and genes from the downregulation cluster in any of the three brain regions were labeled as “downregulation cluster”. Genes were color labeled if they positively or negatively regulate apoptosis according to GO. Genes implicated in both or neither directions by GO terms were not color labeled.

## 3. Results

### 3.1. Most Apoptosis-Related Genes Are Dynamically Regulated in the Developing Forebrain of a Mouse

To characterize expression dynamics of apoptosis-related genes in developing mouse brains, we extracted the expression levels (TPM) of 1923 apoptotic genes in the forebrain region across seven embryonic age points (E10.5, E11.5, E12.5, E13.5, E14.5, E15.5, and E16.5) and one postnatal (P0) age point. Many of these genes are well known for their roles in the classic apoptosis pathway, including caspases, Bcl-2 family proteins, death ligands and receptors, etc. Others are upstream modulators of the classical apoptotic genes, including signaling molecules and transcription factors, etc. The expression values from the ENCODE RNA-seq datasets were log-transformed (log_2_(TPM + 1)) before further Z-score normalization across developmental ages.

**Figure 1:**
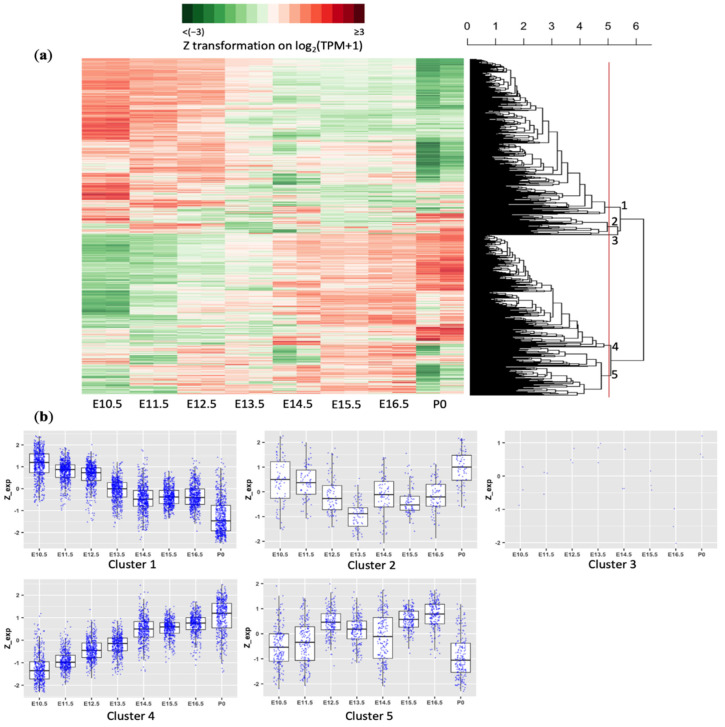
Expression dynamic of apoptosis−related genes during mouse forebrain development: (**a**) Hierarchical clustering and expression heatmap of apoptosis−related genes across development ages (E10.5, E11.5, E12.5, E13.5, E14.5, E15.5, E16.5, P0). Clusters are defined by the red line crossing the dendrogram. Cluster numbers are noted next to each cluster tree. (**b**) mRNA expression of five clusters defined in (**a**). The expression level of each gene was log−transformed (log_2_(TPM + 1)) and then Z−score normalized (i.e., centered by the mean and divided by the standard deviation across different development ages).

A total of 1306 apoptotic genes passed expression filtration (details in Materials and Methods) and were subject to hierarchical clustering, which unbiasedly defined five clusters (Figure 1a). Clusters 1 and 4 were the two major gene groups whose gene expression, respectively, decreased and increased chronologically during brain development (Figure 1b). Cluster 1 contained 603 downregulated apoptotic genes. The median fold change between the last (P0) and the first (E10.5) age points was 0.45 (P0 vs. E10.5). On the contrary, cluster 4 contained 434 apoptotic genes increasing their expression levels with a median 2.45-fold change.

The remaining genes belonged to three smaller clusters. Cluster 2 contained 77 genes, the expression of which were reduced during the early embryonic stage of development but appeared to be upregulated around birth. By contrast, cluster 5 contained 189 genes, modestly increasing expression in the early phase of forebrain development but decreasing expression from E16.5 to P0. Cluster 3 contained only three genes without a clear pattern of temporal regulation. In summary, a majority of the apoptotic genes (1037 out of 1306) falling within cluster 1 or 4 exhibited nearly unidirectional expression changes during mouse forebrain development. We called clusters 1 and 4 the “downregulation cluster” and the “upregulation cluster”, respectively, for further analyses.

### 3.2. Temporal Expression Patterns of Apoptosis-Related Genes Are Consistent across Different Brain Regions during Development

To determine whether the dynamic regulation of these apoptosis-related genes was unique to forebrain development or ubiquitous to general neuronal development, we examined mouse midbrain and hindbrain transcriptomic RNA-seq data from the ENCODE project. Specifically, we determined the temporal expression patterns of the five forebrain clusters during midbrain and hindbrain development. Overall, the temporal patterns for each of these clusters followed a similar trend among the three brain regions (Figure S1). In particular, forebrain cluster 1 displayed a downregulation trend and cluster 4 consistently exhibited an upregulation pattern in both the midbrain and the hindbrain regions.

**Figure 2:**
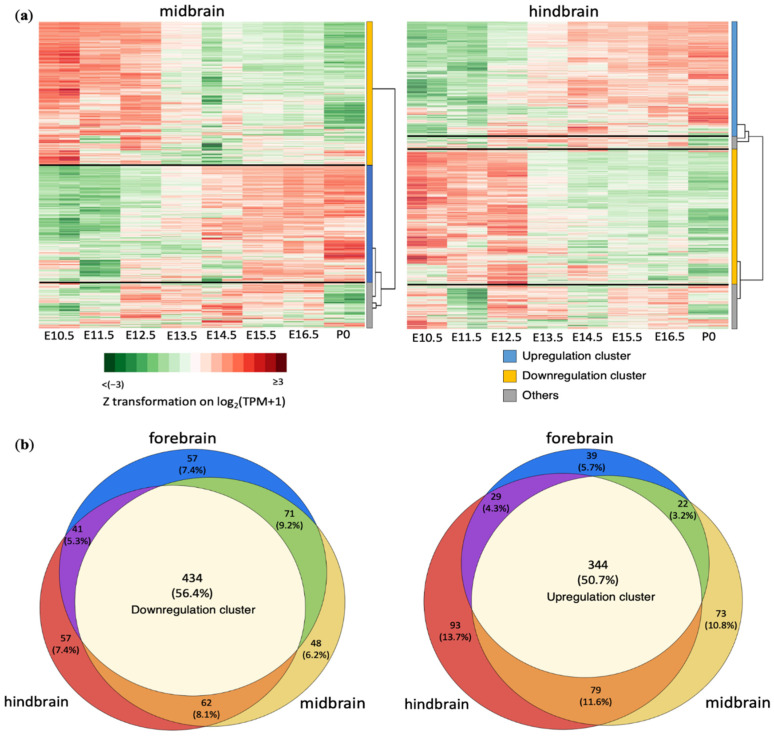
Consistent temporal expression patterns of apoptotic genes during development in three different brain regions: (**a**) Clustering heatmaps of apoptotic gene mRNA expression in the midbrain and hindbrain. Gene clusters with the dendrogram are labeled on the right to the heat maps. (**b**) Venn diagrams of downregulated genes and upregulated genes defined by hierarchical clustering separately performed for three brain regions, showing substantial overlaps among the three brain regions.

We also conducted expression clustering ab initio based on the time-series RNA-seq data from the midbrain and hindbrain regions. As shown in Figure 2a, a majority of apoptotic genes belonged to either a downregulation cluster (615 genes, or 47% of 1317 total expressed genes in the midbrains; 594 genes, or 44% of 1340 total expressed genes in the hindbrains) or an upregulation cluster (518 and 545 genes for midbrains and hindbrains, respectively; or 39% and 41%, respectively, of their total expressed genes).

More importantly, genes in the downregulation clusters largely overlapped between the three brain regions (Figure 2b). A total of 434 downregulated apoptotic genes (56.4% of the cluster union) were identified in all three brain regions. The genes in the upregulation clusters, likewise, shared 344 (50.7%) of apoptotic genes among the three regions. Genes in the upregulation and downregulation clusters of these three brain regions are listed in Table S2. This shows that the regulation of apoptotic genes during neurogenesis is largely consistent across brain regions, indicating their robust transcriptional reprogramming is intrinsic to neuronal differentiation and ubiquitous to neuronal types.

### 3.3. The Regulation of the Classical Apoptosis Pathway during Mouse Brain Development

**Figure 3:**
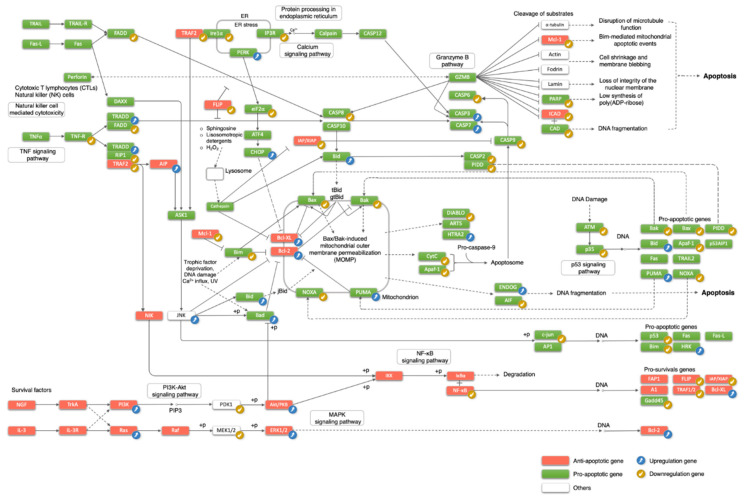
The classic apoptosis pathway from the KEGG database marked with regulatory directions of developmental gene expression changes and functional activity on apoptosis.

We focused on genes in the classic apoptosis pathway, defined by the KEGG database, for their expression dynamics during brain development. These genes were most extensively reported for their roles in apoptosis, whereas other GO-annotated apoptotic genes were studied in a more limited context, for example, in a specific cell type or under a specific treatment condition. As shown in Figure 3, out of the 79 classic apoptosis genes, 39 genes displayed a decreasing expression trend during development in any of the brain regions, while 22 genes increased their expression chronologically. The large proportion of genes changing expression (61/79 or 77%) demonstrates, again, that differentiating neurons drastically alter their regulation of apoptosis during development.

Among the pro-apoptotic genes, 28 were downregulated, outnumbering the 13 upregulated genes, consistent with the notion of decreasing apoptosis competence during neuronal differentiation. The downregulated pro-apoptotic genes included genes in the TNF signaling pathway (*TNF-R*, *FADD*, *RIP1* (*RIPK1*)), genes in the ER stress pathway (*IRE1α* (*ERN1*), *IP3R* (ITPR1), *Eif2α*), pro-apoptotic Bcl-2 family members (*Bax*, *Bak1*, *Noxa* (*PMAIP1*), *Bim* (*BCL2L11*)), and caspases (*Casp2*, *Casp6*, *Casp8*, *Casp9*). Mitochondrial proteins cytochrome C, Diablo, and AIF (*AIFM1*), and DNA damage response genes (ATM, *p35* (*CDK5R1*), *p53* (*TP53*)) also reduced their expression during development.

Among the anti-apoptotic genes, eight were upregulated, including anti-apoptotic Bcl-2 family proteins (*Bcl-2*, *Bcl-xL* (*BCL2L1*)) and the PI3k-Akt signaling pathway (*PI3K* (*PIK3CA*), *Ras* (*HRAS/NRAS/ KRAS*), *Akt* (*AKT1*), *Erk1/2* (*MAPK3/1*)). On the other hand, nine genes were downregulated, including *Traf2*, *Flip* (*CFLAR*), *Xiap*, *Mcl-1*, *Icad* (*DFFA*), and *NFкb* (*NFKB1/2*).

In summary, both anti- and pro-apoptotic genes can be up- or downregulated, but nearly half of the developmentally regulatory events in the classical apoptosis pathway are the downregulation of pro-apoptotic genes, which makes up the largest regulatory gene group (28/(28 + 13 + 8 + 9) = 48%). Interestingly, in the downregulation cluster, classical pro-apoptotic genes were more downregulated than anti-apoptotic genes (fold-change medians: 0.38 vs. 0.67; *p*-value of the one-sided Wilcoxon signed-rank test: 0.04). Therefore, by number or magnitude, the pro-apoptotic genes are more downregulated than the anti-apoptotic genes.

### 3.4. Distinct Histone Modifications Underly the Developmental Regulation of Apoptotic Genes

**Figure 4:**
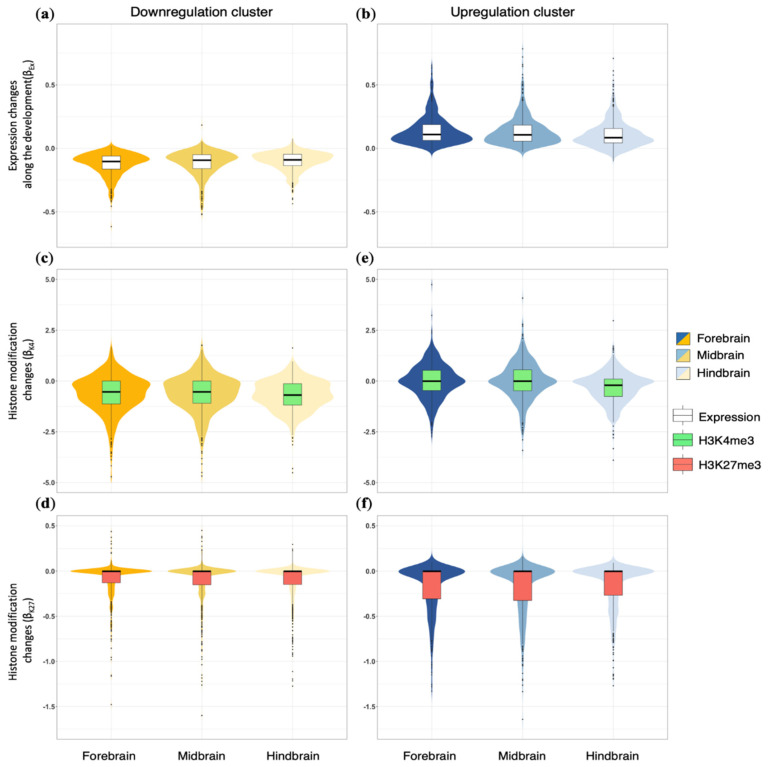
Distinct histone modifications are responsible for the chronical change in expression of the downregulation and upregulation clusters. Regression coefficients (β_Ex_) between mRNA expression and ages for the downregulation clusters (**a**) and the upregulation clusters (**b**) in the forebrain, midbrain, and hindbrain. Regression coefficients (β_K4_) between H3K4me3 signals and ages for genes in the downregulation clusters (**c**) and the upregulation clusters (**d**) in the forebrain, midbrain, and hindbrain. Regression coefficients (β_K27_) between H3K27me3 signals and ages for genes in the downregulation clusters (**e**) and the upregulation clusters (**f**) in the forebrain, midbrain, and hindbrain. All three brain regions show consistent patterns.

To further quantify the temporal expression trends of apoptotic genes, we conducted regression fitting between the gene expression level of each gene and time points (see the Materials and Methods section for details) to derive its regression coefficient of gene expression (β_Ex_) across development. The sign (+ or −) of a coefficient indicates the direction of the developmental change, whereas the absolute value of the coefficients indicate the “speed”, or the “magnitude of the developmental change per time unit”. As expected, the regression coefficients β_Ex_ were negative for the downregulation clusters and positive for the upregulation clusters in all three brain regions (Figure 4a,b).

To understand the mechanisms regulating the developmental expression dynamics of apoptotic genes, we investigated the temporal changes of histone mark H3K4me3, which is known to activate transcription and is highly enriched in gene promoter regions near transcriptional start sites [37]. We also examined repressive histone mark H3K27me3, which also resides around promoter areas but causes downregulation of nearby genes. We utilized the ENCODE ChIP-seq data corresponding to the same developmental ages and the same brain regions for precise inference. For each gene, we obtained the histone mark intensities at its promoter and quantified their changes along the brain development period through a regression fitting between histone mark intensities and time points (see Materials and Methods for details). The regression coefficients (β_K4_, β_K27_) resulting from this analysis represented the change of histone modification intensities over a time unit.

For the downregulation clusters, we found the H3K4me3 coefficients β_K4_ mostly agreed with the developmental gene expression trends. Over three-quarters of genes displayed a negative β_K4_, consistent with the notion that H3K4me3 is positively correlated with transcription (Figure 4c). For these genes, the loss of H3K4me3 at promoters during development implicates reduced transcription or expression levels over time. Interestingly, the repressive H3K27me3 mark showed insignificant contributions to the expression changes, as the median β_K27_ hovered around 0 and most values were relatively small (Figure 4d). In other words, the H3K27me3 patterns do not change much for most genes in the downregulation clusters.

The upregulation clusters exhibited notably different patterns of β_K4_ and β_K27_ than the downregulation clusters. If H3K4me3 alteration was the major driving force of expression changes, the upregulation cluster was expected to contain mostly positive β_K4_. However, the H3K4me3 mark was not accordant with the expression changes, as median β_K4_ was about 0 for the forebrain and midbrain regions (Figure 4e). The hindbrain even atypically exhibited negative β_K4_ for most upregulated genes (median: −0.19). Therefore, H3K4me3 is not predictive of the developmental increase in expression of most apoptotic genes in the upregulation clusters.

By contrast, the loss of repressive H3K27me3 paralleled the increase in apoptotic gene expression. The β_K27_ of the upregulation cluster clearly displayed asymmetric regulatory directions, with over three-quarters of them in a negative range and the others in a negligibly positive range (Figure 4f). This meant, for most genes, peak intensities of H3K27me3 dropped over the developmental period. Taken together, the developmental upregulation of apoptotic genes can be attributed to the consistent removal of H3K27me3 marks from the promoters and, for only a selection of genes, to the addition of H3K4me3 marks.

### 3.5. Developmental Remodeling of H3K4me3 and H3K27me3 Marks Underscores Expression Changes of Pidd1, Casp6, Mapk8, and Tradd

To illustrate the different epigenetic regulations as driving factors of developmental gene expression changes, we examined representative genes from the KEGG apoptotic pathway (Figure 3). For example, *Pidd1* (P53-induced death domain protein 1) and *Casp6* are two genes in the downregulation clusters of all three brain regions. PIDD1, caspase-2, and adaptor protein CRADD form a multiprotein complex, PIDDosome, that activates caspase-2 to initiate apoptosis [41,42]. CASP6 belongs to the cysteine-aspartic acid protease (caspase) gene family and is important for the execution phase of cell death [6,7,8].

**Figure 5:**
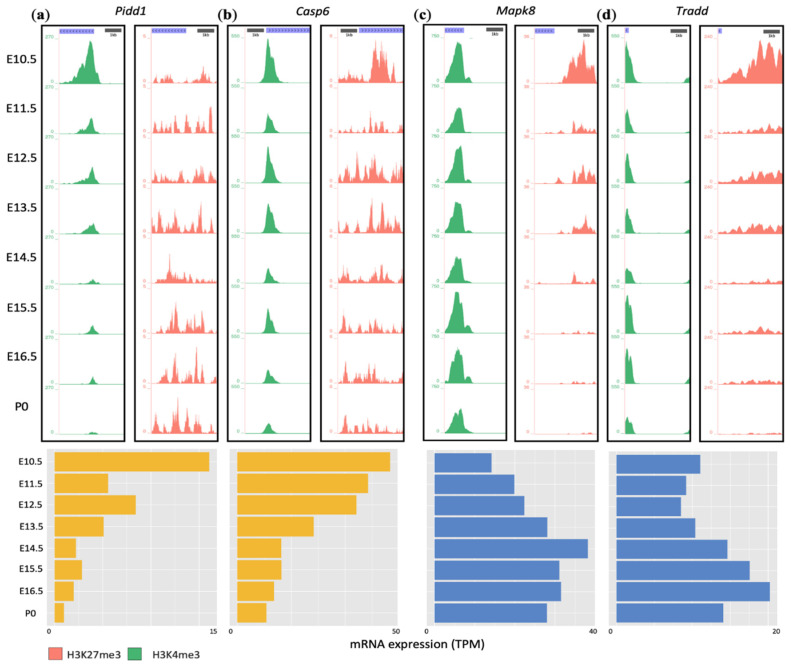
Histone modification patterns of representative genes in developing mouse brain tissues. Top: H3K4me3 (green) and H3K27me3 (red) ChIP-seq peak signals around selected TSSs at eight time points; bottom: mRNA expression of the gene in the same corresponding brain region as shown on the top, analyzed by RNA-seq. (**a**) *Pidd1* in the midbrain, (**b**) *Casp6* in the midbrain, (**c**) *Mapk8* in the forebrain, (**d**) *Tradd* in the forebrain.

As shown in Figure 5a,b, the H3K4me3 peaks at the promoter regions of *Pidd1* and *Casp6* diminished over time. By contrast, the H3K27me3 marks exhibited no pattern of regulation. As a result, *Pidd1* and *Casp6* mRNA expressions were downregulated 9- and 5-fold from E10.5 to P0 in the brain, respectively (Figure 5a,b). Notably, the H3K4me3 peak of *Pidd1* showed the largest drop between E10.5 and E11.5, consistent with the steepest decline in mRNA expression at the same time. Therefore, changes in H3K4me3 rather than H3K27me3 are the main contributor to developmental downregulation of *Pidd1* and *Casp6*.

The upregulation cluster included *Mapk8* and *Tradd* (Figure 5c,d). *Mapk8* encodes mitogen-activated protein kinase 8, belonging to the MAP kinase family [43]. MAPK8 plays an important role in TNF alpha-mediated cell death and UV-induced apoptosis [44,45,46]. *TRADD* encodes TNFR1-associated death domain protein, which functions in the TNFR1 signaling pathway to regulate TNFα-induced extrinsic apoptosis and proteasomal stress-induced apoptosis [47,48,49,50,51]. For both genes, there were no clear trends of developmental changes in H3K4me3. However, their H3K27me3 signals markedly declined over time (Figure 5c,d). This led to an overall increase in mRNA expression of these two genes during development.

## 4. Discussion

Neurons contain a unique regulatory program for apoptosis, as cell death needs to be attenuated to allow continuous neuronal survival. The regulatory program, as regards to apoptotic gene expression, is presumably determined during neuronal development. We found most apoptosis-related genes, including those in the classic apoptosis pathway, are subject to developmental regulation, consistent with the idea that neurons transform how they regulate apoptosis during differentiation. This developmental reprogramming is conserved, sharing most of the upregulated and downregulated genes between different brain regions (forebrain, midbrain, and hindbrain), and, therefore, is intrinsic to the process of neural differentiation and ubiquitous to various neuronal types. Interestingly, neither the up- nor downregulatory trend is restricted to one category of anti- or pro-apoptotic genes (determined by GO terms). This is probably not surprising given the vast number of apoptosis-related genes considered in the study.

However, the core genes in the classical apoptosis pathway (defined by KEGG) exhibit skewed regulation. Specifically, among the pro-apoptotic genes, more are downregulated than upregulated. Among the downregulated genes, pro-apoptotic genes reduce expression more than anti-apoptotic genes do. Notably, while anti-apoptotic Bcl-2 family proteins (*Bcl-2* and *Bcl-xl*) are upregulated, most pro-apoptotic Bcl-2 family proteins (*Bax*, *Bak, Noxa*, *Bim*) and caspases (*Casp2*, *Casp6*, *Casp8*, *Casp9*) are downregulated. This would reduce the overall “fuel” for apoptosis, agreeing with the notion that the balance and relative ratio of pro- and anti-apoptotic genes determine cell death outcomes and that neurons attenuate apoptosis during development [15,16].

*Casp3* and *Casp7* are the exceptions, as they increase expression during embryonic development. They are best known as the executioner caspases of apoptosis for destruction of cellular proteins and structures, irrespective of upstream death signals. In neurons, caspase-3 is also important for dendritic trimming and synaptic pruning, which is necessary during the early postnatal period to establish robust and responsive neural circuits [52,53,54,55,56]. Furthermore, caspase-3 has non-apoptotic functions for synaptic plasticity in adult neurons [57,58]. How this localized function of caspase-3 is activated and restricted without inducing a full-blown cell death program is an interesting subject. The essential role of caspase-3 for neural circuit formation likely restrains its developmental downregulation, or even requires its upregulation so as to be distributed broadly in distant dendritic processes.

Despite upregulation of effector caspases *Casp3* and *Casp7*, neurons uniformly turn down initiator caspases (*Casp2*, *Casp8*, and *Casp9*). This constitutes an effective strategy to attenuate apoptosis, because, in healthy cells, caspases are zymogens with weak or little protease activity and a hierarchy of caspase activation is needed to amplify the destructive signals leading to apoptosis [7,59,60,61]. For example, cytochrome c, released from mitochondria, interacts with Apaf1 to form apoptosome, a complex that recruits caspase-9, causing its self-cleavage and activation, which in turn cleaves and activates caspase-3. Cytochrome c, *Apaf1*, and *Casp9* all decrease mRNA expression during development (Figure 3), effectively reducing the chance of caspase-3 activation.

Another regulatory checkpoint restricting apoptosis is the Bcl-2 family of proteins. Many apoptotic signals converge on BAX and BAK1 in mitochondria by inducing their oligomerization or formation of mitochondrial transition pores, leading to mitochondrial membrane permeabilization and release of cytochrome c and DIABLO [62,63]. BCL-xL and BCL-2 can interact with BAX and BAK1 to inhibit mitochondrial membrane permeabilization [64,65]. Intracellular ratios between anti- and pro-apoptotic BCL-2 family proteins determine cells’ responsiveness to death stimuli [66,67]. This ratio is magnified by the upregulation of *Bcl-xl* and *Bcl-2* and simultaneous downregulation of *Bax* and *Bak1*, thereby significantly diminishing neurons’ vulnerability towards apoptosis activation. Notably, *Bak1* is subject to further post-transcriptional regulation. Increased splicing of *Bak1* exon 5 during development generates an alternative isoform, degraded by the nonsense-mediated mRNA decay pathway without productive translation, such that the BAK1 protein is nearly absent in neurons [24]. The abatement of apoptosome components and the increasing ratio of anti- to pro-apoptotic Bcl-2 family members present two critical “choke” points to attenuate apoptosis in neurons.

The developmental regulation of apoptotic genes coincides with widespread changes in histone modification marks at their promoter regions. This developmental rearrangement of H3K4me3 and H3K27me3 for apoptotic genes may constitute a larger epigenome program. The H3K4me3 landscape continuously changes during brain development and has been implicated in directing neuronal lineage differentiation [38,68,69]. H3K27me3 regulation has also been shown to orchestrate several aspects of neurogenesis, including the balance of self-renewal and differentiation of neural progenitors, as well as the switch from neurogenesis to gliogenesis [70,71,72,73].

We found the upregulation and downregulation clusters of apoptotic genes to undergo distinct dynamic histone modification mark transitions. The predominant driving force for the developmental downregulation of apoptotic genes is reducing H3K4me3 signals on their promoters where H3K7me3 does not significantly change. By contrast, downregulation of H3K27me3 is responsible for developmental upregulation of apoptotic genes, while changes in H3K4me3 are not predictive of the increased gene expression. The differential re-modeling of H3K4me3 and H3K27me3 during development for the upregulation vs. downregulation clusters suggests divergent epigenetic regulatory mechanisms of apoptotic genes, which is an interesting topic for future investigation.

## Data Availability

All datasets used in this study can be accessed at the ENCODE portal (http://encodeproject.org, accessed on 1 October 2021), using the accession IDs provided in Table S3.

## Supplementary Materials

The following are available online at https://www.mdpi.com/article/10.3390/cells10112901/s1, Figure S1: Expression of apoptotic genes in midbrain and hindbrain regions. Table S1: GO terms related to apoptosis. Table S2: Apoptotic genes in the downregulation and upregulation cluster in the three brain regions. Table S3: Accession IDs of RNA-seq and CHIP-Seq data analyzed in this study.

## References

[1] Galluzzi L., Vitale I., Aaronson S.A., Abrams J.M., Adam D., Agostinis P., Alnemri E.S., Altucci L., Amelio I., Andrews D.W. (2018). Molecular mechanisms of cell death: Recommendations of the Nomenclature Committee on Cell Death 2018. Cell Death Differ..

[2] Green D.R., Llambi F. (2015). Cell Death Signaling. Cold Spring Harb. Perspect. Biol..

[3] Elmore S. (2007). Apoptosis: A review of programmed cell death. Toxicol. Pathol..

[4] Peter M.E. (2011). Programmed cell death: Apoptosis meets necrosis. Nature.

[5] Green D.R., Kroemer G. (2004). The pathophysiology of mitochondrial cell death. Science.

[6] McIlwain D.R., Berger T., Mak T.W. (2015). Caspase functions in cell death and disease. Cold Spring Harb. Perspect. Biol..

[7] Van Opdenbosch N., Lamkanfi M. (2019). Caspases in Cell Death, Inflammation, and Disease. Immunity.

[8] Larsen B.D., Sørensen C.S. (2017). The caspase-activated DNase: Apoptosis and beyond. FEBS J..

[9] Fricker M., Tolkovsky A.M., Borutaite V., Coleman M., Brown G.C. (2018). Neuronal Cell Death. Physiol. Rev..

[10] Dekkers M.P., Barde Y.A. (2013). Developmental biology. Programmed cell death in neuronal development. Science.

[11] Yamaguchi Y., Miura M. (2015). Programmed cell death in neurodevelopment. Dev. Cell.

[12] Williams R.W., Herrup K. (1988). The control of neuron number. Annu. Rev. Neurosci..

[13] Kristiansen M., Ham J. (2014). Programmed cell death during neuronal development: The sympathetic neuron model. Cell Death Differ..

[14] Huang E.J., Reichardt L.F. (2001). Neurotrophins: Roles in neuronal development and function. Annu. Rev. Neurosci..

[15] Kole A.J., Annis R.P., Deshmukh M. (2013). Mature neurons: Equipped for survival. Cell Death Dis..

[16] Benn S.C., Woolf C.J. (2004). Adult neuron survival strategies--slamming on the brakes. Nat. Rev. Neurosci..

[17] Easton R.M., Deckwerth T.L., Parsadanian A.S., Johnson E.M. (1997). Analysis of the mechanism of loss of trophic factor dependence associated with neuronal maturation: A phenotype indistinguishable from Bax deletion. J. Neurosci..

[18] Davies A.M. (1998). Developmental changes in the neurotrophic factor survival requirements of peripheral nervous system neurons. Prog. Brain Res..

[19] Spreafico R., Frassoni C., Arcelli P., Selvaggio M., De Biasi S. (1995). In situ labeling of apoptotic cell death in the cerebral cortex and thalamus of rats during development. J. Comp. Neurol..

[20] Blaschke A.J., Staley K., Chun J. (1996). Widespread programmed cell death in proliferative and postmitotic regions of the fetal cerebral cortex. Development.

[21] Blaschke A.J., Weiner J.A., Chun J. (1998). Programmed cell death is a universal feature of embryonic and postnatal neuroproliferative regions throughout the central nervous system. J. Comp. Neurol..

[22] Thomaidou D., Mione M.C., Cavanagh J.F., Parnavelas J.G. (1997). Apoptosis and its relation to the cell cycle in the developing cerebral cortex. J. Neurosci..

[23] McConnell M.J., MacMillan H.R., Chun J. (2009). Mathematical modeling supports substantial mouse neural progenitor cell death. Neural Dev..

[24] Lin L., Zhang M., Stoilov P., Chen L., Zheng S. (2020). Developmental Attenuation of Neuronal Apoptosis by Neural-Specific Splicing of Bak1 Microexon. Neuron.

[25] Petito C.K., Olarte J.P., Roberts B., Nowak T.S., Pulsinelli W.A. (1998). Selective glial vulnerability following transient global ischemia in rat brain. J. Neuropathol. Exp. Neurol..

[26] Chow B.M., Li Y.Q., Wong C.S. (2000). Radiation-induced apoptosis in the adult central nervous system is p53-dependent. Cell Death Differ..

[27] Casafont I., Palanca A., Lafarga V., Berciano M.T., Lafarga M. (2011). Effect of ionizing radiation in sensory ganglion neurons: Organization and dynamics of nuclear compartments of DNA damage/repair and their relationship with transcription and cell cycle. Acta Neuropathol..

[28] Li Y.Q., Guo Y.P., Jay V., Stewart P.A., Wong C.S. (1996). Time course of radiation-induced apoptosis in the adult rat spinal cord. Radiother. Oncol..

[29] Labi V., Erlacher M. (2015). How cell death shapes cancer. Cell Death Dis..

[30] Hanahan D., Weinberg R.A. (2000). The hallmarks of cancer. Cell.

[31] Lomvardas S., Maniatis T. (2016). Histone and DNA Modifications as Regulators of Neuronal Development and Function. Cold Spring Harb. Perspect. Biol..

[32] Kouzarides T. (2007). Chromatin modifications and their function. Cell.

[33] Zhang T., Cooper S., Brockdorff N. (2015). The interplay of histone modifications—Writers that read. EMBO Rep..

[34] Kimura H. (2013). Histone modifications for human epigenome analysis. J. Hum. Genet..

[35] Voigt P., Tee W.W., Reinberg D. (2013). A double take on bivalent promoters. Genes Dev..

[36] Chen L. (2010). A link between H3K27me3 mark and exon length in the gene promoters of pluripotent and differentiated cells. Bioinformatics.

[37] Liang G., Lin J.C., Wei V., Yoo C., Cheng J.C., Nguyen C.T., Weisenberger D.J., Egger G., Takai D., Gonzales F.A. (2004). Distinct localization of histone H3 acetylation and H3-K4 methylation to the transcription start sites in the human genome. Proc. Natl. Acad. Sci. USA.

[38] Cheung I., Shulha H.P., Jiang Y., Matevossian A., Wang J., Weng Z., Akbarian S. (2010). Developmental regulation and individual differences of neuronal H3K4me3 epigenomes in the prefrontal cortex. Proc. Natl. Acad. Sci. USA.

[39] Ku M., Koche R.P., Rheinbay E., Mendenhall E.M., Endoh M., Mikkelsen T.S., Presser A., Nusbaum C., Xie X., Chi A.S. (2008). Genomewide analysis of PRC1 and PRC2 occupancy identifies two classes of bivalent domains. PLoS Genet..

[40] Zhu L.J., Gazin C., Lawson N.D., Pagès H., Lin S.M., Lapointe D.S., Green M.R. (2010). ChIPpeakAnno: A Bioconductor package to annotate ChIP-seq and ChIP-chip data. BMC Bioinform..

[41] Tinel A., Tschopp J. (2004). The PIDDosome, a protein complex implicated in activation of caspase-2 in response to genotoxic stress. Science.

[42] Tinel A., Janssens S., Lippens S., Cuenin S., Logette E., Jaccard B., Quadroni M., Tschopp J. (2007). Autoproteolysis of PIDD marks the bifurcation between pro-death caspase-2 and pro-survival NF-kappaB pathway. EMBO J..

[43] Parra E., Gutiérrez L., Ferreira J. (2015). Inhibition of basal JNK activity by small interfering RNAs enhances cisplatin sensitivity and decreases DNA repair in T98G glioblastoma cells. Oncol. Rep..

[44] Sabio G., Davis R.J. (2014). TNF and MAP kinase signalling pathways. Semin. Immunol..

[45] Liu J., Minemoto Y., Lin A. (2004). c-Jun N-terminal protein kinase 1 (JNK1), but not JNK2, is essential for tumor necrosis factor alpha-induced c-Jun kinase activation and apoptosis. Mol. Cell Biol..

[46] Tournier C., Hess P., Yang D.D., Xu J., Turner T.K., Nimnual A., Bar-Sagi D., Jones S.N., Flavell R.A., Davis R.J. (2000). Requirement of JNK for stress-induced activation of the cytochrome c-mediated death pathway. Science.

[47] Baker E., Chen L.Z., Smith C.A., Callen D.F., Goodwin R., Sutherland G.R. (1991). Chromosomal location of the human tumor necrosis factor receptor genes. Cytogenet Cell Genet..

[48] Pobezinskaya Y.L., Kim Y.S., Choksi S., Morgan M.J., Li T., Liu C., Liu Z. (2008). The function of TRADD in signaling through tumor necrosis factor receptor 1 and TRIF-dependent Toll-like receptors. Nat. Immunol..

[49] Chen N.J., Chio I.I., Lin W.J., Duncan G., Chau H., Katz D., Huang H.L., Pike K.A., Hao Z., Su Y.W. (2008). Beyond tumor necrosis factor receptor: TRADD signaling in toll-like receptors. Proc. Natl. Acad. Sci. USA.

[50] Michallet M.C., Meylan E., Ermolaeva M.A., Vazquez J., Rebsamen M., Curran J., Poeck H., Bscheider M., Hartmann G., König M. (2008). TRADD protein is an essential component of the RIG-like helicase antiviral pathway. Immunity.

[51] Xu D., Zhao H., Jin M., Zhu H., Shan B., Geng J., Dziedzic S.A., Amin P., Mifflin L., Naito M.G. (2020). Modulating TRADD to restore cellular homeostasis and inhibit apoptosis. Nature.

[52] Hyman B.T., Yuan J. (2012). Apoptotic and non-apoptotic roles of caspases in neuronal physiology and pathophysiology. Nat. Rev. Neurosci..

[53] D’Amelio M., Cavallucci V., Cecconi F. (2010). Neuronal caspase-3 signaling: Not only cell death. Cell Death Differ..

[54] Hollville E., Deshmukh M. (2018). Physiological functions of non-apoptotic caspase activity in the nervous system. Semin. Cell Dev. Biol..

[55] Gulyaeva N.V. (2003). Non-apoptotic functions of caspase-3 in nervous tissue. Biochemistry.

[56] Geden M.J., Romero S.E., Deshmukh M. (2019). Apoptosis versus axon pruning: Molecular intersection of two distinct pathways for axon degeneration. Neurosci. Res..

[57] Li Z., Sheng M. (2012). Caspases in synaptic plasticity. Mol. Brain.

[58] Chan S.L., Mattson M.P. (1999). Caspase and calpain substrates: Roles in synaptic plasticity and cell death. J. Neurosci. Res..

[59] Goyal L. (2001). Cell death inhibition: Keeping caspases in check. Cell.

[60] Nuñez G., Benedict M.A., Hu Y., Inohara N. (1998). Caspases: The proteases of the apoptotic pathway. Oncogene.

[61] Cohen G.M. (1997). Caspases: The executioners of apoptosis. Biochem. J..

[62] Jürgensmeier J.M., Xie Z., Deveraux Q., Ellerby L., Bredesen D., Reed J.C. (1998). Bax directly induces release of cytochrome c from isolated mitochondria. Proc. Natl. Acad. Sci. USA.

[63] Narita M., Shimizu S., Ito T., Chittenden T., Lutz R.J., Matsuda H., Tsujimoto Y. (1998). Bax interacts with the permeability transition pore to induce permeability transition and cytochrome c release in isolated mitochondria. Proc. Natl. Acad. Sci. USA.

[64] Hardwick J.M., Soane L. (2013). Multiple functions of BCL-2 family proteins. Cold Spring Harb. Perspect. Biol..

[65] Czabotar P.E., Lessene G., Strasser A., Adams J.M. (2014). Control of apoptosis by the BCL-2 protein family: Implications for physiology and therapy. Nat. Rev. Mol. Cell Biol..

[66] Chipuk J.E., Moldoveanu T., Llambi F., Parsons M.J., Green D.R. (2010). The BCL-2 family reunion. Mol. Cell.

[67] Youle R.J., Strasser A. (2008). The BCL-2 protein family: Opposing activities that mediate cell death. Nat. Rev. Mol. Cell Biol..

[68] Shulha H.P., Cheung I., Guo Y., Akbarian S., Weng Z. (2013). Coordinated cell type-specific epigenetic remodeling in prefrontal cortex begins before birth and continues into early adulthood. PLoS Genet..

[69] Burney M.J., Johnston C., Wong K.Y., Teng S.W., Beglopoulos V., Stanton L.W., Williams B.P., Bithell A., Buckley N.J. (2013). An epigenetic signature of developmental potential in neural stem cells and early neurons. Stem Cells.

[70] Pereira J.D., Sansom S.N., Smith J., Dobenecker M.W., Tarakhovsky A., Livesey F.J. (2010). Ezh2, the histone methyltransferase of PRC2, regulates the balance between self-renewal and differentiation in the cerebral cortex. Proc. Natl. Acad. Sci. USA.

[71] Hirabayashi Y., Suzki N., Tsuboi M., Endo T.A., Toyoda T., Shinga J., Koseki H., Vidal M., Gotoh Y. (2009). Polycomb limits the neurogenic competence of neural precursor cells to promote astrogenic fate transition. Neuron.

[72] Sparmann A., Xie Y., Verhoeven E., Vermeulen M., Lancini C., Gargiulo G., Hulsman D., Mann M., Knoblich J.A., van Lohuizen M. (2013). The chromodomain helicase Chd4 is required for Polycomb-mediated inhibition of astroglial differentiation. EMBO J..

[73] Egan C.M., Nyman U., Skotte J., Streubel G., Turner S., O’Connell D.J., Rraklli V., Dolan M.J., Chadderton N., Hansen K. (2013). CHD5 is required for neurogenesis and has a dual role in facilitating gene expression and polycomb gene repression. Dev. Cell.

