# Supplementary material for: Global Reprogramming of Apoptosis-Related Genes during Brain Development
Source: Cells. 2021 Oct 27;10(11):2901. doi: 10.3390/cells10112901 (PMC8616463; doi:10.3390/cells10112901)
Supplement: Supplementary file 1 [file cells-10-02901-s001.zip › cells-1415850-supplementary/cells-1415850 -SI/SupplementaryFigureS1.pdf]

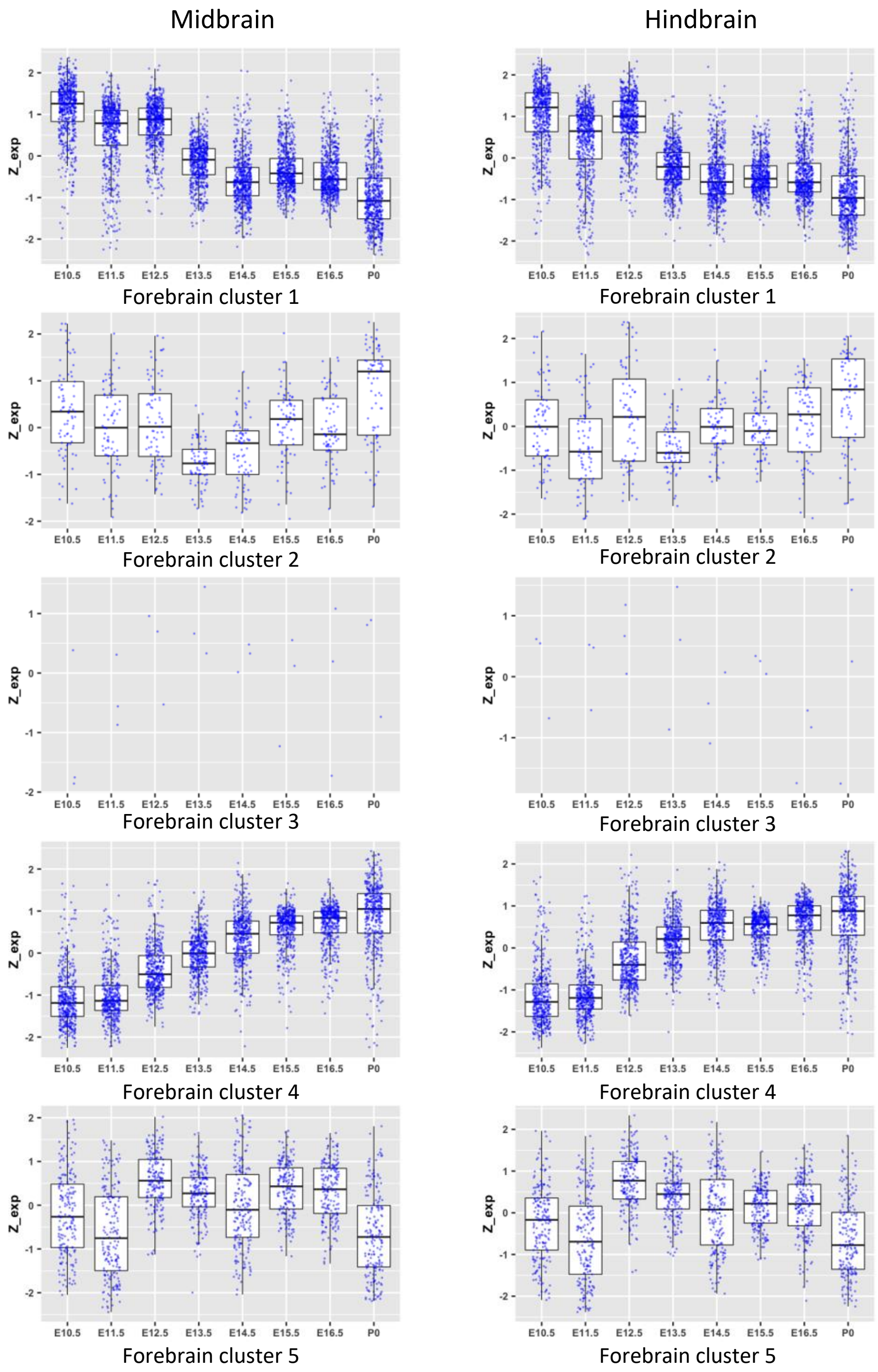

Figure S1. Expression of apoptotic genes in midbrain and hindbrain regions. Gene clusters were defined by the forebrain data.
